# The Reporting of Racehorse Fatalities in New Zealand Thoroughbred Flat Racing in the 2011/12–2021/22 Seasons

**DOI:** 10.3390/ani13040612

**Published:** 2023-02-09

**Authors:** Michaela J. Gibson, Kylie A. Legg, Erica K. Gee, Chris W. Rogers

**Affiliations:** 1 School of Veterinary Sciences, Massey University, Palmerston North 4442, New Zealand; 2 School of Agriculture and Environment, Massey University, Private Bag 11-222, Palmerston North 4442, New Zealand

**Keywords:** Thoroughbred racing, steward, race day fatality, cardiac failure, catastrophic musculoskeletal injury

## Abstract

**Simple Summary:**

Race day fatalities, associated with fracture and suspected cardiac failure, represent a significant welfare concern. To reduce race day fatalities, data are needed to determine the risk factors under New Zealand racing conditions. The aim of this study was to examine race and horse-level risk factors for race day fatalities in New Zealand Thoroughbred flat racing using retrospective race day data from the 2011/12–2021/22 racing seasons. Horse and race-level factors associated with fatality within 72 h of racing were identified by merging fatality data with master race data for the corresponding seasons. The incidence of fatalities was low and most fatalities were the result of euthanasia due to fatal fracture. Horses which raced over greater distances were more likely to sustain a fatal fracture than horses racing ≤1600 m. Male horses and firmer track conditions were also associated with an increase in the risk of fatal fracture. Horses aged 5 years and older were more likely to die from heart failure than younger horses. The shift in the reporting system to an online app resulted in a greater level of detail provided for fatality events, enabling the identification of specific risk factors.

**Abstract:**

Race day fatalities as a consequence of catastrophic musculoskeletal injury and cardiac failure are both a welfare concern and provide a challenge for the social perceptions of equine welfare within the racing industry. To reduce race day fatalities, the risk factors under New Zealand racing conditions need to be identified. The aim of this study was to examine race and horse-level risk factors for fatalities in New Zealand Thoroughbred flat racing using retrospective race day data from the 2011/12–2021/22 racing seasons. Horse and race-level factors associated with a suspected cardiac failure and fatal fracture were identified by merging fatality data with the master race dataset for the corresponding seasons. Most fatalities were associated with fatal fracture (0.4 per 1000 starts, 95% CI 0.4–0.5). Horses which raced over distances > 1600 m were 1.7 times (95% CI 1.2–2.5) more likely to sustain a fatal fracture than horses racing ≤ 1600 m. Male horses and firmer track conditions were also associated with an increase in the risk of fatal fracture. Horses aged 5 years and older were 2.1 (95% CI 1.1–4.6) times more likely to suffer a suspected cardiac failure than younger horses. Changes in the industry reporting system improved the level of detail provided for fatalities, enabling the identification of specific risk factors.

## 1. Introduction

Raceday horse fatalities are a significant welfare concern for racing industries worldwide. In addition, race day horse fatalities undermine the social perceptions of equine welfare within the racing industry [1]. To address welfare concerns and reduce horse fatalities in racing, the possible risk factors specific to New Zealand need to be identified. The two most common causes of race day fatality are catastrophic musculoskeletal injuries resulting in euthanasia and sudden death from cardiac failure [2].

There is variation in the incidence rates of horse fatality reported in the international literature due to case definitions and differences in racing structure and rules between jurisdictions [3]. As examples, within the literature low rates of race day fatality associated with musculoskeletal injury were reported in Victoria, Australia (0.33 per 1000 starts) [4], in Great Britain (0.6–0.8 per 1000 starts) [5], whilst in the USA, individual studies and the pooled study incidence rate is greater at (1.62 per 1000 starts,) [3]. The recent trends for the USA are for reducing race day fatality incidence rates, but the values remain above the pooled incidence rate of catastrophic musculoskeletal injuries over multiple countries and jurisdictions, as calculated by Hitchens, et al. [3], which was reported to be 1.2 per 1000 starts.

In recognition of the importance to document race day racehorse injury and providing consistency in case definition and reporting processes, there have been changes in practice in most of the international racing jurisdictions. The introduction of the equine injury database for horse racing in the USA represented a major step towards uniformity of reporting and collation of data [6].

Previous New Zealand failure to finish data was analysed prior to 2011 and included all fatal and non-fatal fractures and cardiac and respiratory events. The incidence rate for all musculoskeletal injuries was 0.7 per 1000 starts whilst the incidence of cardiac and respiratory events was 0.21 per 1000 starts [7]. The incidence of fatal musculoskeletal injuries was reported as 0.4 per 1000 starts [7]. Since the publication of this data the Racing Integrity Board (RIB, the official racing compliance organisation for all three racing codes in New Zealand, previously called the Racing Integrity Unit) assumed the management of race day reporting. This included the reporting of race day events in the form of stipendiary stewards reports and clinical outcomes of veterinary examinations [7]. Data collected after the introduction of the RIB and new online reporting systems resulted in greater detail describing race day events [8]. During the 2014/2015 racing season, the RIB restricted race day regulatory veterinarians to a cohort of contracted equine veterinarians, as opposed to the previous model where race day veterinarians were recruited from the local veterinary practice. In addition, an incident report form was implemented to aid the standardisation of veterinary examinations on race day [7]. Although data integrity was improved, the use of a paper-based system continued to result in some errors as a result of manual entry such as misspelt horse names or variation in terms used to describe a race day event and clinical outcome [9]. In the 2019/20 racing season, an online app-based reporting system for stipendiary stewards’ reports was introduced into both harness and Thoroughbred racing in New Zealand. The online app was adapted from the Australian Racing Incident Database (ARID) and utilised a sequence of drop-down prompts to provide consistent descriptors of an event and the anatomical descriptors and clinical outcomes associated with the resultant veterinary examination [10].

Risk factors for race day fatalities have been reported for a number of racing jurisdictions. However, there are variations between jurisdictions in the magnitude and nature of the risk factors for race day fatalities [3,9]. To optimise strategies to reduce race day fatalities, it is therefore important to examine the risk factors specific to a racing jurisdiction. Therefore, the aim of this study was to examine the race and horse-level risk factors for race day fatalities in New Zealand Thoroughbred flat racing.

## 2. Materials and Methods

Data were obtained for all flat racing starts during the 2011/12–2021/22 racing seasons as a Microsoft Excel (Microsoft Corporation, Redmond, WA, USA) spreadsheet from New Zealand Thoroughbred Racing Inc (the official registration body for Thoroughbred racing in New Zealand). The output contained the racing conditions (track type, track condition, race length, race time, number of starters, etc.) and horse information (name, age and sex). In addition, an Excel spreadsheet including all race day fatalities for the same racing seasons, as recorded by the race day stipendiary stewards was obtained from the RIB. The race day fatality output recorded the date, racecourse, race number, horse name, fatality type, descriptor of the event and the associated clinical outcome.

### 2.1. Definitions

The case definition was all horses that died or were euthanised during a race meeting or which died or were euthanised within 72 h of competing in a race (in the seasons 2017/18–2021/22), as a result of catastrophic musculoskeletal injury (CMI) or suspected cardiac failure. These horses were examined by the race day veterinarian and the findings were reported on the veterinarian report, or via the online recording app in the seasons 2019/20 to 2021/22 [10]. Clinical findings, anatomical sites affected and euthanasia were officially reported in the race day stewards’ reports [10]. A CMI was defined as a muscle, tendon or bone injury that resulted in death or euthanasia within 72 h of the race. These were further stratified by fatal fractures and fatal soft tissue injuries. Post-mortem examinations are not routinely carried out on race day fatalities.

Euthanasia in the 72-h period after racing was the responsibility of the trainers’ treating veterinarian and is required to be officially declared under the New Zealand rules of racing since the commencement of the 2017/18 racing season. Horses that did not compete in a race or competed in a race that was abandoned were excluded from the analysis.

### 2.2. Statistical Analysis

#### 2.2.1. Data Cleaning

All data cleaning was performed by one researcher (M.J.G). To obtain information about the racing surface condition, race class, horse age, number of races the horse completed and other track and horse information, the race day fatality output was merged with the official race start records and results. Apparent errors, such as misspelt horse names and incorrect dates were checked manually against the official formal transcript of the relevant stipendiary steward’s report, archived on the New Zealand Thoroughbred Racing website. Track conditions were defined based on the current track rating system as “firm” (penetrometer reading 1.0–2.0) “good” (penetrometer reading 3.0–4.0), “soft” (penetrometer reading 5.0–7.0), “heavy” (penetrometer reading 8–10) and synthetic (synthetic track surface). Race distance was categorised as sprint (≤1400 m), mile (1401–1799 m), middle distance (1800 m–2099 m) and staying (>2100 m). There was limited variation in number of starters per race (11 IQR 9–13) [11], therefore this variable was categorised into less than 9 or 9 or more starters per race. Horse age was initially examined as categories of 2, 3, 4 and ≥5 years old but was subsequently collapsed to a category of less than 5 and ≥5 years old to examine the impact of the right shift in the domestic horse racing population age profile reported by Legg, et al. [11].

A variable for recording systems was generated to reflect reporting of veterinary records, where fatalities were recorded by race day veterinarians provided by the local veterinary clinic from 2011/12 to 2013/14 (‘local veterinarian’); following the introduction of contracted race day veterinarians from 2014/15 to 2018/19 (‘contracted veterinarian’); and with the implementation of the online system from 2019/20 to 2021/22 (‘online system’). The sex of the horse was defined as either male (stallion, colt, gelding or rig) or female (mare or filly). A rig was a horse that was a cryptorchid. Jockey experience was defined as either apprentice or jockey. An apprentice was defined as any rider with a weight reduction of >1 kg from the handicap weight assigned to the horse.

#### 2.2.2. Data Analysis

The distribution of median race starts by recording system and age for suspected cardiac failure, fatal fracture and fatal soft tissue injury were examined using a Kruskall–Wallis test. Incidence rates for an event occurring were described as the number of events per 1000 starts with 95% confidence intervals (95% CI) [12]. Differences in the incidence rates for the cause of death between recording systems were examined using a Wald test.

The incidence rate ratio of the occurrence of a suspected cardiac failure and fatal fracture and 95% CI at a univariable level were calculated for the following variables; recording system, age category (≤5 and >5 years old), sex (male [and female), number of starters in the race (<9 and ≥9), race distance (≤1600 m and >1600 m), track condition (firm, good, dead, soft, heavy, and synthetic), track direction (anticlockwise and clockwise), jockey weight (<54 kg, 54- < 56 kg, 56- < 58 kg and ≥58 kg) and jockey experience (apprentice and jockey) using a Poisson regression in a generalised linear mixed model. Variables were screened in univariable models, and if obtaining the significance threshold (*p* < 0.2), were included in the multivariable level. The multivariable models were built using a backwards selection procedure whereby variables that improved the model, based on a chi-squared likelihood ratio test (*p* ≤ 0.05), were retained in the model. Horse, as a random effect was tested as a null model and did not improve the model fit so was removed from the analysis. No risk factor analysis was conducted for the fatal soft tissue injuries due to their low incidence and heterogeneous nature.

All statistical analyses were conducted using RStudio (version 3.5.1, 2018; R Foundation for Statistical Computing, Vienna, Austria) with a level of significance set at *p* < 0.05.

## 3. Results

### 3.1. Population

There were 297,293 starts over the 2011/12–2021/22 flat racing seasons of which 91,028 (30.6%) starts were in the 2011/12–2013/14 seasons, 135,545 (45.6%) were in the 2014/15–2018/19 seasons and 70,720 (23.8%) were in the 2019/20–2021/22 seasons. There were 26,925 horses that had a start in at least one season. The majority of starts were held on turf tracks (27,773 races, 99.2%) of which the majority of the races were held on “good” (11,834 races, 42.3%) or “heavy” tracks (8763 races, 31.3%). The remaining races held on a turf track were on “soft” (6381 races, 22.8 %), and “firm” (795 races, 2.8%). Races held on synthetic tracks only accounted for 0.8% (212 races) of all races. The majority of starts were run in an anticlockwise direction (220,288 starts, 74.1%) with fewer in a clockwise direction (77,005 starts, 25.9%). The median race distance was 1400 m [IQR 1200–1600 m] with 22.9% (6409/27,985) of races being over a mile (1600 m). There was a median of 11 starters in a race [IQR 9–13] with a median starter age of 4 years old [IQR 4–5]. Two-year-old horses started in fewer races per season with a median of 2 starts [IQR 1–3] per season, followed by three-year-olds with 4 starts [IQR 2–6] and ≥4 year olds with 6 starts [IQR 3–9] (*p* < 0.05).

Half of the starts were by geldings (50.3%), followed by mares and fillies (48.2%) and colts, stallions and rigs (1.5%).

### 3.2. Causes of Fatalities

There were 174 race day fatalities in flat racing over the 11 seasons (0.6 per 1000 starts, 95% CI 0.5–0.7). Race day fatalities that occurred on the racecourse grounds, but occurred prior to the race start or the race was abandoned were removed from the data (*n* = 6, 2 cardiovascular, 3 fatal fractures and 1 for a severe laceration). The remaining dataset contained 168 fatalities over the 11 seasons with 32 coded as suspected cardiac failure (0.1 per 1000 starts, 95% CI = 0.07–0.2), 128 coded as fatal fracture (0.4 per 1000 starts, 95% CI = 0.4–0.5) and 8 coded as fatal soft tissue injury (0.03 per 1000 starts, 95% CI = 0.01–0.05).

The median age for fatalities was 5 years old [IQR 4–6] and did not differ between the cause of deaths *p* = 0.779).

There was no effect of recording systems on the incidence rate of suspected cardiac failure, fatal fracture and fatal soft tissue injury (Table 1).

#### 3.2.1. Suspected Cardiac Failure

Of the suspected cardiac failures, 28 (87.5%) resulted in sudden death and the other 4 (12.5%) required the horse to be euthanised. At a univariable level, horses aged 5 or over were 2.1 times more likely to sustain a suspected cardiac failure than horses aged 4 and under (Table 2). There was no association with the recording system, number of participants, track condition/surface, horse sex, race distance, weight carried and jockey experience on the incidence of suspected cardiac failures. There were no suspected cardiac failure events on “firm” and “synthetic” tracks, so races held on these tracks were omitted in the univariable model for track condition. As only one factor was significant, a multivariable analysis was not performed.

#### 3.2.2. Fatal Fracture

Of the fatal fractures, 126 (98.4%) resulted in euthanasia on race day and the remaining 2 (1.6%) were euthanised within 48 h of the race. The majority of fatal fractures occurred in races in an anticlockwise direction (91/220,288 starts, 70.5% of fractures) with fewer fatal fractures in a clockwise direction (38/77,005 starts, 29.5% of fractures). However, the incidence of fractures in each track direction reflected the distribution of starts in each direction (*p* > 0.05). Most horses that were reported to sustain a fatal fracture did not finish the race (*n* = 102, 79.7%) due to the horse being pulled up (*n* = 76, 59.4%). The remaining fatal fractures (20.3%) were associated with the horse falling (*n* = 19, 14.8%) or the jockey falling (*n* = 7, 5.5%).

The majority of fatal fractures were in the appendicular skeleton (Table 3), with forelimbs (62.8% of fractures) having the highest proportion of fractures followed by hind limbs (26.4% of fractures). Fatal fractures in the axial skeleton contributed only 3.1% of fatal fractures and all were recorded as involving the pelvis (*n* = 4). Overall, 22% of reports for fatal fractures resulting in euthanasia did not state which bone was fractured, 11% did not state which limb was affected and 3.1% did not identify the bone or limb affected. One horse fractured both the left humerus and left metatarsal. The local veterinarian recording system (seasons 2011/12–2013/14) had the greatest proportion of fractures classified as unknown within the season (11/28, 39.3%), followed by the contracted veterinarian group (15/63, 23.8%) with the lowest proportion of unclassified fractures associated with the introduction of the online reporting system (2/37, 5.4%).

There was an association of recording system, number of starters, track condition, horse age, horse sex, race distance and weight carried with the incidence of fatal fractures at a univariable level (Supplementary Table S1). In the multivariable model, track condition, horse sex and race distance had an association with the incidence of fatal fractures requiring euthanasia (Table 4). Male horses were 1.7 times more likely to sustain a fatal fracture than females. Horses racing in sprint races were less likely to sustain a fatal fracture. Starters racing on a “heavy” track were less likely to sustain a fatal fracture compared to good tracks.

## 4. Discussion

The overall incidence rate for race day fatalities (0.6 per 1000 starts, 95% CI 0.5–0.7) in New Zealand was similar to values reported for Australia and lower than jurisdictions, such as Great Britain (0.7–0.8 per 1000 starts), the USA (2.5 per 1000 starts) [13] and a recent study reporting racing in Ontario, Canada (1.8 per 1000 starts) [14]. There is a large movement of racehorses and similarity in the patterns of racing and training between Australia and New Zealand [15], and thus the similarity of incidence rates was not surprising. The use of equine veterinarians and the online system did not alter the incidence of reporting and the incidence of fatalities reported but did improve the detail of the reporting of fatalities. For example, early reports for fatal fractures stated that a fracture had occurred but often did not state the affected bone and/or the limb. By having a greater level of detail and consistency in terms used to describe the anatomical location and events leading to fatality, the associated risk factors can be determined for specific sites or presentations.

The incidence of suspected cardiac failure (0.1 per 1000 starts, 95% CI = 0.07–0.2) was similar to Great Britain (0.08 per 1000 starts) [16] and Australia (0.08 per 1000 starts) [17]. The precise identification of the cardiac event associated with race day fatalities is often difficult, even with a post-mortem examination. Most reports provided the caveat of ”suspected“ cardiac failure or rupture in the clinical notes field. A post-mortem examination may not have dramatically improved the precision in identifying the precise event, given that the results of a multicentre study identified that only 53% of race day sudden deaths were given a definitive diagnosis by the attending pathologist [18].

In agreement with Lyle, et al. [19], older horses appear to be at greater risk of race day cardiac failure. Conversely, Nath, et al. [2] suggested that horses vulnerable to sudden cardiac failure are more likely to die early in their career. However, Nath’s study had a different case definition and was based on horses that were submitted for post-mortem and included horses that died during training, which contributed to 75% of horse fatalities as a result of cardiac failure. The inclusion of horses in training, as well as race day events meant that horses that died at a lower exercise intensity were included [2]. By including horses that died from cardiac failure in training, horses that had a pre-existing condition and did not race were included. Many horses enter race training as a 2-year-old but do not have an official race until they are a 3-year-old, which may also be a contributing factor for the lower reported median age for cardiac failure of 3.6 years [2] compared to the current study that reported a median of 5 years.

The incidence rate of fatal fractures requiring euthanasia has remained constant over the past eleven seasons. In some cases of fatal fractures, there may be latent diagnosis where lame horses are taken home and examined by the trainer’s own veterinarian the following day or when the horse fails to become sound [20]. It is at this time, that radiographs are often taken, and a fracture can be confirmed. Fractures of this nature are not displaced and can often present as lameness which progressively deteriorates as the horse cools down from the race or in the days following the race. Prior to 2017, these fractures that resulted in euthanasia after race day were not required to be reported to the race day regulatory body (stipendiary stewards). In 2017, a rule was established, stating that all horses that died or were euthanised within 72 h of racing must be reported. Extension of the reporting period of up to 72 h after race day conform with current international reporting practices and enables a comprehensive dataset of race day fatalities to be established. Therefore, the number of race day fatalities, as a result of fractures, would be expected to increase. The lack of increase in the number of fatalities, after the introduction of the rule, may suggest that the majority of euthanasia cases associated with fractures occur on race day. Fractures that are identified post-race day are possibly less severe, leading to an attempt at rehabilitation or rehoming attempts.

The incidence of race day fatal fractures was similar to previous reports in Australia (0.4 per 1000 starts), Great Britain (0.6 per 1000 starts) and Hong Kong (0.6 per 1000 starts) [16,21,22], and lower than the USA (1.6–1.8 per 1000 races) [3,6]. The lower incidence of fatal fractures compared to the USA may be associated with differences in racing and track conditions, such as the use of dirt tracks and claiming races [3].

The majority of fatal fractures occurred in the forelimb, in particular in the metacarpus and proximal phalanx. Fractures at these sites are often associated with stress, where repetitive loading of the bone causes microdamage and increases the likelihood of catastrophic failure [20]. Fracture risk increases with age as a result of bone fatigue [23]. Bone fatigue can occur due to high volumes of loading under the monotonic force threshold for catastrophic failure which causes microscopic damage in a localised area where the force is placed. The accumulation of microdamage increases fracture risk over time [24].

Horses participating in longer races were at greater risk of a fatal fracture. This may be due to greater time at risk due to longer races and greater load cycles and a greater possibility of fatigue [4]. Longer races, and the possibility of greater time at risk, is in agreement with findings from previous New Zealand studies reporting risk factors for musculoskeletal injury and other racing jurisdictions, where racing distances over a mile increases the risk of musculoskeletal injury [7] and fracture [4,23].

Firmer tracks are recognised as a risk factor for musculoskeletal injuries due to the lack of cushioning resulting in a greater force exerted on limbs as the hoof impacts the ground [7,25]. In the current study few races were conducted on firm tracks (2.8%), and though the incidence of fatal fractures was greater when compared to those conducted on good tracks, this did not reach statistical significance. In New Zealand track managers actively avoid fast going with the use of irrigation to ensure most races are run with a penetrometer reading of 3 (“good” track condition) [26]. Stipendiary stewards’ also examine the track prior to race day to avoid running race meetings on tracks that are not conducive to horse welfare and jockey safety. Heavy tracks had a lower risk of fatal fracture than firmer tracks, possibly due to slower race speeds, and thus lower relative strain rate [25], associated with these conditions. In addition, during the months in winter with the heaviest rainfall, races tend to be run on tracks that have adequate drainage to provide consistent race conditions [27].

A greater risk of fatal fractures in male horses in some racing jurisdictions has been proposed to be due to the greater muscle mass and bodyweight of male horses in comparison to their female counterparts [4]. However, within the New Zealand system, the increased incidence associated with male fatal fracture risk may be due to the fact that most male horses in New Zealand are geldings and there is preferential retirement of fillies/mares to breeding [11,28]. Retired mares may be utilised for breeding purposes, and New Zealand has a large Thoroughbred breeding and export industry [29]. In comparison, geldings have no latent reproductive potential and are more likely to be retained in racing for longer [4]. Therefore, the New Zealand industry is confounded as the majority of the older horses in the racing population are more likely to be geldings. However, in recent years, the proportion of females racing in the older population has been increasing, possibly due to fewer mares being repurposed as broodmares [11].

Jockey safety and horse safety are inter-related and CMI often results in both horse and jockey falls. The proportion of horse and jockey falls associated with a CMI or fatal fracture in New Zealand was similar to that reported in California and in Australia (New South Wales and Australian Capital Territory) with approximately 20% of fatal fractures associated with jockey falls [13,21]. The relative distribution of the location of the fracture was also similar across studies, with the majority associated with the distal forelimb. Considering that most fractures occurred in the distal forelimb, it is surprising that the frequency of jockey falls is not higher. This data indicates that in many situations, jockeys may be detecting subtle changes in gait immediately prior to the onset of fracture that permits them to take evasive action and attempt to “pull up” the horse. In the United Kingdom, races restricted to non-professional jockeys were reported to pose a higher risk for horses to experience race day injuries [25]. However, within the ranks of professional jockeys, there may be less variation in riding ability and associated risk of injury or fatal fracture. In the current results, there was no effect of jockey experience on the risk of fatal fracture. This result is in agreement with other studies [4,7], and is possibly due to the low incidence of musculoskeletal injuries and limited variation in riding ability within the New Zealand jockey ranks [30].

A limitation of the current study was that information about the affected horses was restricted to what was recorded within the racing record, i.e., age, sex, racing starts. Information regarding the horse’s training regime and environmental factors were unknown, but play a role in the risk of a horse having a fatal race day event [20]. Retrospective horse level training data suitable for epidemiology study is not readily available in many racing jurisdictions, including New Zealand. Data on cumulative exercise load can be inputted from race history, such as cumulative starts, pattern of starts (preparations and lengths of spells) and starts within a defined timeframe. The current study reported horse and race level variables associated with race day fatalities and did not include variables associated with the pattern of racing, which will be considered in a subsequent study. The administration of medications, either on or close to race day have been associated with increased risk of musculoskeletal injury [31]. There is limited data published on medication practices during training with racehorses in New Zealand, which prevented examination of this as a risk factor. However, in New Zealand there are extensive restrictions on the use of ergogenic and ergolytic substances effectively making Thoroughbred racing drug-free [32].

The current dataset only considered fatalities that occurred on race day and more recently in the 72 h after. Horses that have underlying heart conditions or underlying injury may sustain a catastrophic event in training and have an incident prior to a race day start [2]. This is reflected in the observations from other studies that racehorses were more likely to suffer a sudden death event during training than on race day and at least half of fatal fractures occur during training [2,20,33]. Based on the amount of time spent training, versus the amount of time racing, it would be expected that the incidence of fatalities would be much higher at training due to greater time at risk. However, the majority of training is carried out at a lower intensity than racing, reducing the risk of a fatality event [15].

Within the current study, the descriptors for the fatalities were based on clinical findings recorded during the veterinarian examination, and in some cases the presumptive diagnosis, without additional diagnostic investigations or post-mortem examination [5]. As a result, clinical findings in some reports were vague. In the case of cardiac failures, many sudden deaths would be classified as cardiac failure due to the lack of other obvious clinical reasons for death. A post-mortem examination may provide additional information on the cause of the sudden death, such as exercise-induced pulmonary haemorrhage (epistaxis). However, some post-mortem findings will still conclude cardiac failure as a result due to the exclusion of other possible reasons [2]. In some musculoskeletal fracture cases, early reports only stated the limb or gross anatomical location. Without radiographs or post-mortem examination, it is difficult to identify the precise location and aetiology of the fracture. Prospectively, the improved detail recorded via the online app and the industry fatality post-mortem scheme should generate robust data, permitting the identification of risk factors with specific events associated with race day fatality.

## 5. Conclusions

The incidence of race day fatality in New Zealand was similar to countries, such as Great Britain and Australia. Older horses had a greater likelihood of a cardiac failure. Most fatal fractures occurred in the distal limb. Male horses and increased race distance were associated with a greater incidence of fractures resulting in euthanasia, whilst heavy tracks were associated with reduced incidence. During the observation period, there was an increasing level of detail recorded for fatality events, reflecting proactive changes in the industry regulatory reporting practices. Both the improved level of recording and the industry race day fatality post-mortem scheme, should generate more descriptive data, enabling more in-depth identification of risk factors for specific events associated with race day fatalities. These data can then be used to implement evidence-based regulatory changes and prospectively monitor the effectiveness of any changes.

## Figures and Tables

**Table 1:** Incidence rates and 95% confidence intervals for suspected cardiac failures, fatal fractures, and fatal soft tissue injuries by recording system for the 2011/12–2021/22 Thoroughbred flat racing seasons.

	Local Veterinarian 2011/12–2013/14 (*n* = 91,028)		Contracted Veterinarian 2014/15–2018/19 (*n* = 135,545)		Online System 2019/20–2021/22 (*n* = 70,720)		*p*-Value
Cause of Death	*n*	Incidence	*n*	Incidence	*n*	Incidence	
Suspected cardiac failure	10	0.1 [0.06–0.2]	17	1.1 [0.08–0.2]	5	0.07 [0.03–0.2]	0.530
Fatal fracture	29	0.3 [0.2–0.4]	63	0.4 [0.4–0.6]	37	0.5 [0.4–0.7]	0.115
Fatal soft tissue injury	2	0.01 [0.002–0.06]	4	0.04 [0.02–0.09]	2	0.03 [0.01–0.1]	0.951

**Table 2:** Univariable incidence rate ratios (IRR) and 95% confidence of a suspected cardiac failure occurring with the effects of the recording system, number of participants, track surface, horse age, horse sex and race distance for Thoroughbred flat racing in the 2011/12–2021/22 seasons.

Univariable	Cardiac *n* (%)	Starts	IRR [95% CI]	*p*-Value	Wald *p*-Value
Recording system					
Local veterinary clinic	10 (31.3%)	91,028	(Referent)		0.530
Contracted veterinarian	17 (53.1%)	135,545	1.1 [0.5–2.6]	0.747	
Online system	5 (15.6%)	70,720	0.6 [0.2–1.8]	0.416	
Number of starters					
Less than nine	4 (12.5%)	47,123	(Referent)		0.607
Nine or more	28 (87.5%)	250,170	1.3 [0.5–4.4]		
Track condition/surface					
Firm (Turf)	0 (%)	7752	-		
Good (Turf)	16 (50%)	126,852	(Referent)		0.693
Soft (Turf)	8 (25%)	90,985	0.9 [0.4–2.1]	0.838	
Heavy (Turf)	8 (25%)	69,288	0.7 [0.3–1.6]	0.405	
Synthetic	0 (0%)	2180	-		
Horse age					
Under five years	11 (34.4%)	157,361	(Referent)		0.040
Five years and over	21 (65.6%)	139,932	2.1 [1.1–4.6]		
Horse sex					
Male (Colt, gelding stallion)	16 (50.0%)	153,981	0.9 [0.5–1.9]		
Female (Filly or mare)	16 (50.0%)	143,312	(Referent)		0.840
Race distance					
Sprint (≤1400 m)	15 (46.9%)	160,096	1.3 [0.4–8.3]	0.723	
Mile (1401–1799 m)	8 (25.0%)	70,350	1.6 [0.4–1.1]	0.560	
Middle distance (1800–2099 m)	2 (6.3%)	27,877	(Referent)		0.490
Staying (≥2100 m)	7 (21.9%)	38,970	2.5 [0.6–1.7]	0.252	
Weight carried (kg)					
<54	4 (12.5%)	32,879	(Referent)		0.766
54–<56	7 (21.9%)	87,878	0.7 [0.2–2.5]	0.499	
56–<58	14 (43.8%)	108,588	1.1 [0.4–3.7]	0.918	
≥58	7 (21.9%)	67,438	0.9 [0.3–3.3]	0.800	
Jockey experience					
Apprentice	10 (33.3%)	84,394	(Referent)		0.720
Jockey	22 (66.7%)	212,901	0.9 [0.4–1.9]		

**Table 3:** Anatomical site identified with fatal soft tissue injury and fatal fractures associated with a race day fatalities in Thoroughbred flat racing from 2011/12–2021/22 racing seasons.

	Left Fore	Right Fore	Left Hind	Right Hind	Not Specified	Total
Fatal soft tissue injury						
Ligament	1	1		1	1	4 (50.0%)
Tendon	1	2	1			4 (50.0%)
Fatal fracture						
Humerus	8	4			1	13 (10.1%)
Radius	2	1				3 (2.3%)
Radiocarpal		1				1 (0.8%)
Carpus	4	2			1	7 (5.4%)
Metacarpal	16	12			1	29 (22.5%)
Pelvis					4	4 (3.1%)
Femur				2	1	3 (2.3%)
Tibia			1	1		2 (1.6%)
Metatarsal			8	3	1	12 (9.3%)
Proximal phalanx	9	3	4	5		21 (16.3%)
Sesamoid	2	2		1	1	6 (4.7%)
Not specified	12	3	3	6	4	28 (21.7%)
Total	53 (41.1%)	28 (21.7%)	16 (12.4%)	18 (14.0%)	14 (10.9%)	

**Table 4:** Multivariable incidence rate ratios and 95% confidence of a fatal fracture occurring with the effects of track condition/ surface, horse sex and race distance for Thoroughbred flat racing in the 2011/12–2021/22 seasons.

Multivariable	Fractures	Starts	IRR [95% CI]	*p*-Value	Wald *p*-Value
Track condition					
Firm (Turf)	8 (6.3%)	7752	2.0 [0.9–4.0]	0.060	
Good (Turf)	63 (49.2%)	126,852	(Referent)		0.004
Soft (Turf)	34 (26.6%)	90,985	1.0 [0.6–1.5]	0.962	
Heavy (Turf)	22 (17.2%)	69,288	0.5 [0.3–0.8]	0.003	
Synthetic	1 (0.8%)	2180	1.0 [0.1–4.4]	0.976	
Horse sex					
Male (Colt, gelding stallion)	84 (65.1%)	153,981	1.7 [1.2–2.4]	0.006	
Female (Filly or mare)	45 (34.9%)	143,312	(Referent)		0.006
Race distance					
Sprint (≤1400 m)	51 (39.8%)	160,096	0.5 [0.3–0.8]	0.007	
Mile (1401–1799 m)	32 (25.3%)	70,350	0.7 [0.4–1.2]	0.160	
Middle distance (1800–2099 m)	20 (15.6%)	27,877	(Referent)		0.014
Staying (≥2100 m)	25 (19.5%)	38,970	0.9 [0.5–1.7]	0.780	

## Data Availability

Data available from corresponding author upon request.

## Supplementary Materials

The following supporting information can be downloaded at: https://www.mdpi.com/article/10.3390/ani13040612/s1, Supplementary Table S1: Univariable incidence rate ratios (IRR) and 95% confidence interval of a fatal fracture occurring with the effects of season group, number of participants, track surface, horse age, horse sex and race distance for Thoroughbred flat racing in the 2011/12–2021/22 seasons.
