# Supplementary material for: The Reporting of Racehorse Fatalities in New Zealand Thoroughbred Flat Racing in the 2011/12–2021/22 Seasons
Source: Animals (Basel). 2023 Feb 9;13(4):612. doi: 10.3390/ani13040612 (PMC9951738; doi:10.3390/ani13040612)
Supplement: Supplementary file 1 [file animals-13-00612-s001.zip › animals-1967315-supplementary.pdf]

Supplementary Table S1. Univariable incidence rate ratios (IRR) and 95% confidence interval of a fatal fracture occurring with the effects of season group, number of participants, track surface, horse age, horse sex and race distance for Thoroughbred flat racing in the 2011/12-2021/22 seasons.

Univariable	Fractures	Starts	IRR [95% CI]	p- value	Wald p- value
Season group					
Local veterinary clinic	29 (22.5%)	91,028	(Referent)		0.120
Contracted veterinarian	63 (48.8%)	135,545	1.5 [0.9-2.3]	0.096	
Online system	37 (28.7%)	70,720	1.6 [1.0-2.7]	0.047	
Number of starters					
Less than 9	15 (11.6%)	47,123	(Referent)		0.194
9 or more	114 (88.4%)	250,170	1.4 [0.9-2.6]		
Track condition/ Surface					
Firm (Turf)	8 (6.3%)	7,752	2.0 [0.9-4.0]	0.056	
Good (Turf)	63 (49.2%)	126,852	(Referent)		0.005
Soft (Turf)	34 (26.6%)	90,985	1.0 [0.6-1.5]	0.896	
Heavy (Turf)	22 (17.2%)	69,288	0.5 [0.3-0.8]	0.003	
Synthetic	1 (0.8%)	2,180	0.9 [0.1-4.1]	0.924	
Track direction					
Anticlockwise	91 (70.5%)	220,288	(Referent)		0.357
Clockwise	38 (29.5%)	77,005	1.2 [0.8-1.7]		
Horse age					
Under 5	56 (43.4%)	157,361	(Referent)		0.031
5 and over	73 (56.6%)	139,932	1.5 [1.0-2.1]		
Sex					
Male (Colt, gelding stallion)	84 (65.1%)	153,981	1.7 [1.2-2.5]		
Female (Filly or mare)	45 (34.9%)	143,312	(Referent)		0.003
Race distance					
Sprint (≤1400m)	51 (39.8%)	160,096	0.5 [0.3-0.8]	0.003	
Mile (1401-1799m)	32 (25.3%)	70,350	0.6 [0.4-1.1]	0.110	
Middle distance (1800-2099m)	20 (15.6%)	27,877	(Referent)		
Staying (≥2100m)	25 (19.5%)	38,970	0.9 [0.5-1.6]	0.709	
Weight carried (kg)					
< 54	13 (10.1%)	32879	(Referent)		0.062
54-< 56	28 (21.7%)	87878	0.8 [0.4-1.6]	0.520	
56-< 58	47 (36.4%)	108588	1.1 [0.6-2.1]	0.773	
≥58	41 (31.8%)	67438	1.5 [0.8-3.0]	0.176	
Jockey experience					
Apprentice	43 (33.3%)	84394	(Referent)		0.214
Jockey	86 (66.7%)	212901	0.8 [0.6-1.2]		
